# Bone and mineral metabolism in 2–7-year-old Finnish children and their caregivers following vegan, vegetarian, and omnivorous diets

**DOI:** 10.1007/s00394-025-03758-y

**Published:** 2025-09-11

**Authors:** Suvi T. Itkonen, Topi Hovinen, Elina Kettunen, Riitta Freese, Venla Tilli, Kevin D. Cashman, Maijaliisa Erkkola, Anu Suomalainen, Liisa Korkalo

**Affiliations:** 1 Department of Food and Nutrition, University of Helsinki, P.O. Box 66, 00790 Helsinki, Finland; 2 Stem Cells and Metabolism Program, Faculty of Medicine, University of Helsinki, Helsinki, Finland; 3 School of Food and Nutritional Sciences, University College Cork, Cork, Ireland; 4 HiLife, University of Helsinki, Helsinki, Finland; 5 HUS Diagnostics, Helsinki University Hospital, Helsinki, Finland

**Keywords:** Plant-based diet, Vitamin D, Parathyroid hormone, Protein, Calcium, Bone turnover

## Abstract

**Purpose:**

Limited evidence exists on the effects of plant-based diets (PBDs) on bone metabolism, especially in children. We studied bone and mineral metabolism and intakes of bone-related nutrients in 2–7-year-old children and their caregivers who followed vegan, vegetarian (collectively referred to as PBD), and omnivorous diets.

**Methods:**

Blood samples were collected from 29, 18, and 24 children following vegan, vegetarian, or omnivorous diets (mean age 4.5 ± 1.5 years, 51% girls) and from 29, 23, and 24 adult caregivers, respectively (mean age 38.2 ± 4.4 years, 57% women). Serum tartrate-resistant acid phosphatase 5b (S-TRAP5b), bone-specific alkaline phosphatase (S-BAP), 25-hydroxyvitamin D (S-25(OH)D), and plasma parathyroid hormone (P-PTH) were analysed using linear trends by contrast analysis [adjusted for age, sex, and (standardized) body mass index]. Nutrient intakes were calculated from 3-day food records**.**

**Results:**

Linear trends towards higher concentrations of P-PTH among children and bone resorption marker S-TRAP5b and bone formation marker S-BAP among adults in PBDs were observed (*P* < 0.05). Mean vitamin D intakes (diet + supplements) were adequate (> 10 µg/d), mean 25(OH)D concentrations were > 70 nmol/l, vitamin D supplement use was common (74–100%), and mean calcium intakes were approximately adequate in all age and diet groups**.** Protein intake was lower in PBDs (12–14 E%) than in omnivores (16–17 E%) (*P* < 0.05).

**Conclusion:**

Linear trends towards increased bone catabolism among children and accelerated bone turnover among adults following PBDs were observed despite adequate vitamin D status and approximately adequate calcium intake. The role of lower protein intake and calcium bioavailability in PBDs and bone health requires further investigation.

**Supplementary Information:**

The online version contains supplementary material available at 10.1007/s00394-025-03758-y.

## Background

The recently updated Nordic Nutrition Recommendations (NNR) stress that a shift towards predominantly plant-based diets (PBDs) is needed to promote health and environmental sustainability [1], following the framework established by the EAT-Lancet consortium [2]. PBD [here we refer to vegan (VGN) and vegetarian (VGT) diets together] have nutritional benefits and are associated with a lower risk of many chronic diseases such as cardiovascular disease and cancers [3, 4]. However, they may also lead to lower intakes of certain nutrients crucial for bone health, particularly vitamin D and calcium (Ca) [5–7]. Low vitamin D status can lead to lower Ca absorption and secondary hyperparathyroidism, i.e. to increased parathyroid hormone (PTH) concentrations, and further to bone loss [8]. NNR suggest that groups with no or low intake of dairy products are at risk of Ca deficiency and individuals who limit fish products in their diets are at risk of vitamin D deficiency if not consuming fortified foods or supplements [1]. Those following VGN diets are provided as examples for both of these groups [1]. However, a recent systematic review comparing PBD and omnivorous (OMN) diets showed that particularly children and adolescents were at risk of inadequate vitamin D and Ca intakes, regardless of their diet [9].

The current evidence does not completely answer the question of whether the switch towards more PBD benefits or harms bone health. In their systematic review and meta-analysis, including 20 studies with > 37,000 participants, Iguacel et al. [10] showed that VGT and VGN had lower femoral neck and lumbar spine bone mineral density (BMD) and VGN also had higher fracture incidence than OMN. Later, two large UK-based studies, one with > 400,000 and the other with > 26,000 participants, revealed that persons with VGT diet had a higher risk of hip fracture than regular meat-eaters [11, 12]. Moreover, in the EPIC-Oxford cohort study, over a mean follow-up of 17.6 years, non-meat eaters, especially VGN, had higher risks of either total or some site-specific fractures, particularly hip fractures [13]. Studies focusing on bone turnover markers have shown higher concentrations of bone resorption markers or catabolic PTH [14–16] among VGN adults than among OMN adults. However, results on bone formation markers have been inconsistent [14–16].

Suitability of the VGN diet for children has been widely debated, and in Germany, for instance, it has not been recommended for children [17]. Regarding PBD and bone turnover in children, the number of studies is small, and, to the best of our knowledge, there is no previous data comparing children following VGN diet with their OMN peers. However, Polish data comparing VGT and OMN children aged 4–10 years have been published [18–21]. Mostly higher bone resorption marker [18, 20, 21] and PTH [19] concentrations have been observed, but the results for bone formation markers are contradictory [18, 19, 21]. Concerning BMD, VGT children have had lower values than OMN children [18], and in one study [22] the significant difference in bone mineral content (BMC) remained for VGN diet even after adjustment for body size.

Finland has a wide national vitamin D food fortification policy [23], along with supplementation recommendations, particularly for at-risk groups [24]. This has improved the vitamin D situation in the general population over the last decades [25]. Various dairy products are an integral part of the Finnish diet and food culture, serving as a major source of calcium and, when fortified, also vitamin D. Thus, from the bone health perspective it is important to study vulnerable groups, in particular children, who follow PBD and restrict the use of traditional dietary sources of central bone-related nutrients. In this study, we aimed to investigate bone and mineral metabolism as well as intakes of key bone-related nutrients in 2–7-year-old Finnish children and their caregivers following VGN, VGT, or OMN diets.

## Methods

### Design

The cross-sectional MIRA2 study, which focuses on dietary intakes, nutritional status, and metabolomics in children following VGN, VGT, and OMN diets, and their caregivers, was conducted in the public and private early childhood education and care (ECEC) centres in Helsinki, Finland between September 2021 and June 2022. The target group was children aged 2–6 years at time of informed consent (some turned 7 years prior to blood sampling) and their caregivers. The study was conducted according to the guidelines laid down in the Declaration of Helsinki, and written informed consent was obtained from adult participants for their own participation and from each child’s legal guardian for the child’s participation. To ensure that children understood the study procedures, a link to a video with informed consent information appropriate for approximately 5–6-year-old children was also provided to the families. All procedures were approved by the Ethics Committee of the Helsinki and Uusimaa Hospital District (2061/2021).

We contacted the municipal ECEC centres with at least one child following a VGN diet, where the personnel delivered the invitation letters and a link to an initial interest electronic form to families via email. In each centre, recruitment was conducted in two phases: first, children following a VGN diet were recruited, and then children following VGT or OMN diets from the same ECEC centre, respectively. Also the caregivers were invited to participate, regardless of their diet. In March 2022, the recruitment was expanded to include the private ECEC centres to reach the target number of subjects recruited.

### Sample size estimation

In the present bone-related study, we used biological samples and data from the MIRA2 study, the main aim of which was to investigate the nutritional status and nutrient intakes of children following VGN, VGT, or OMN diets. Within the boundaries regarding the number of ECEC centres permitted to be contacted and based on our experience from a previous study [26], we estimated that we could reach 40 participants per diet group. This was in line with the MIRA2 study’s power calculations, which indicated that 30 participants per group would be sufficient to show a statistical difference with a 95% CI and a statistical power of 0.80 based on 0.73 standard deviation in a set of primary clinical variables of interest (serum retinol, vitamin E, and ferritin). No sample size calculations for bone turnover or mineral metabolism were carried out since this was a secondary research question within the MIRA2 study.

### Exclusion criteria and sample size

**Fig. 1:**
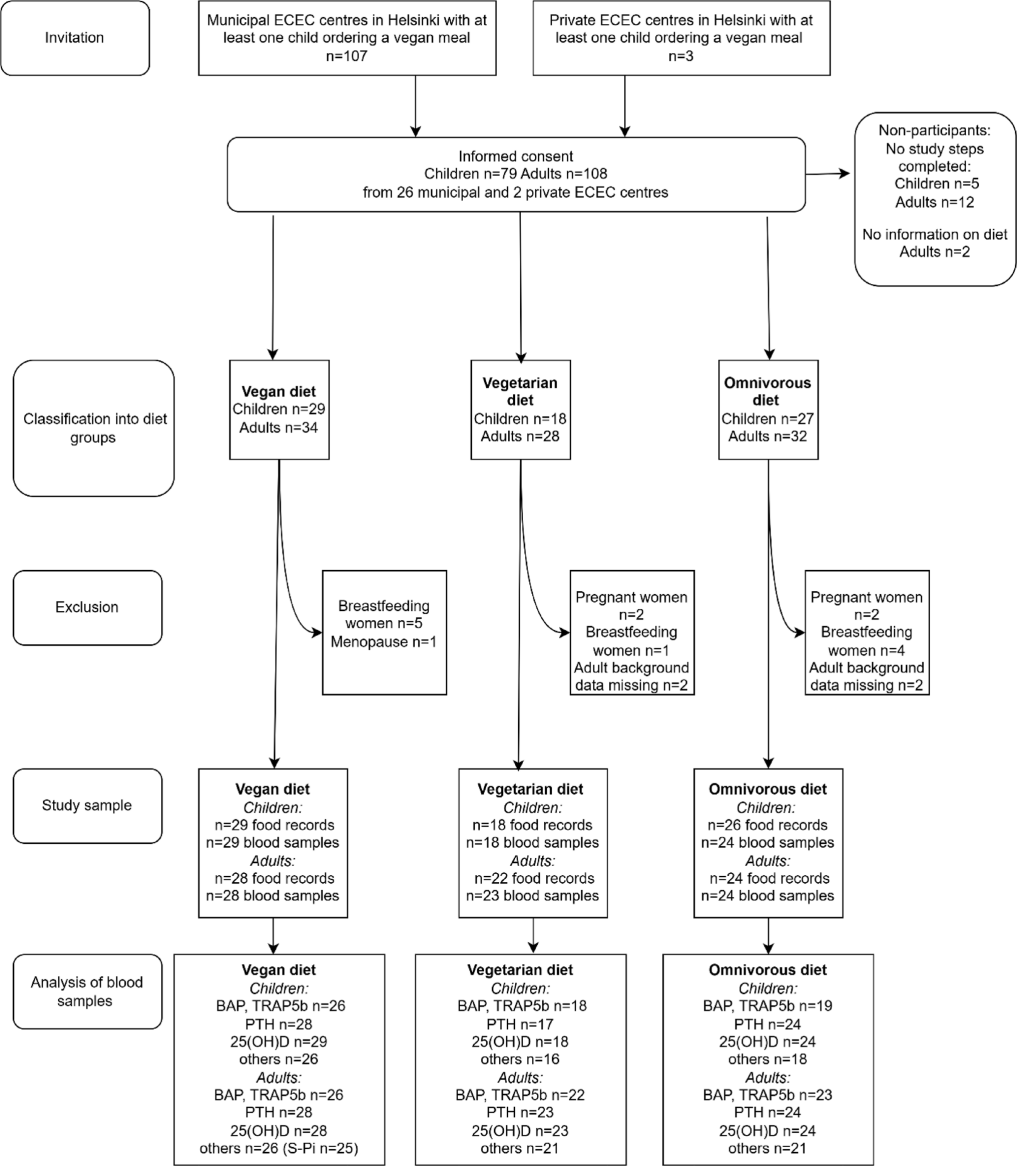
Enrolment of the MIRA2 study. ECEC, early childhood care and education; PTH, parathyroid hormone; S-25(OH)D, serum 25-hydroxyvitamin D; S-ALP, serum total alkaline phosphatase; S-BAP, bone-specific alkaline phosphatase; S-Ca, serum calcium; S-Pi, serum phosphate; S-TRAP5b, serum tartrate-resistant acid phosphatase 5b

Exclusion criteria for children were regular medication (not allergy medication), gluten-free diet, diabetes, extensive food allergies, breastfeeding of the child during the study, and body weight < 10 kg. The exclusion criterion for adult participants was living with the participating child for less than 50% of the time. The flow diagram (Fig. 1) shows the enrolment of the study. Altogether 79 children and 108 caregivers from 28 ECEC centres (26 public and two private) in Helsinki gave informed consent to participate in the study. The final numbers of participants, i.e. subjects whose diet information was available (see Background data and diet groups), were 74 children and 94 adults. Of the children, 29 followed a VGN diet, 18 a VGT diet, and 27 an OMN diet. For the caregivers, the numbers were 34, 28, and 32, respectively. Altogether 10 sibling pairs participated in the study. From this bone-related sub-study, pregnant (n = 4), breastfeeding (n = 10), and menopausal (n = 1) women were excluded due to potential effects on bone turnover. In addition, four adult subjects without background data were excluded.

### Background data and diet groups

Research Electronic Data Capture (REDCap) platform (version 11.1.18, Vanderbilt University) was used to collect background data of the participants by questionnaires. Caregivers filled in the questionnaire on behalf of the children. The questionnaire included questions on e.g. diet, age, duration of the current diet, use of medications, and dietary supplements. For adults, additional questions covered weight, height, education, physical activity, smoking, alcohol consumption, and whether they were pregnant or breastfeeding at the time of the study. We assigned the participants to VGN, VGT, and OMN groups based on responses in the background questionnaire. Those who reported following a VGT diet containing fish, eggs, and/or dairy products were assigned to a VGT diet group. The background questionnaires (with a question on the followed diet) for two children were missing, and they were assigned to OMN diet group according to manually examined food records (FR) (n = 1), or in case of missing FR (n = 1) according to initial interest form containing information on diet. Originally, four adults with missing background questionnaires were assigned to diet groups based on their FR but they were excluded from this bone-related substudy due to lack of covariate of information provided by the questionnaire.

Adult participants recorded their major type of leisure-time physical activity through a multiple-choice question with predefined active hours per week. They also recorded alcohol consumption based on the frequency of consumption and number of servings consumed, e.g. one serving (12 g of 100% alcohol) corresponds to a bottle (0.33 l) of medium-strength beer, 120 ml of wine, or 40 ml of spirits. The frequency of use and dose of dietary supplements consumed during the last month including brand names were recorded for children and adults. For children, weight was measured using a Seca 878 Mobile flat scale or a Seca 704 Column scale and height was measured with a Seca 213 portable stadiometer. Standard deviation score for body mass index (SDS-BMI) was calculated based on the Finnish population growth data [27]. For adults, BMI was calculated [weight (kg)/height (m^2^)] based on the information provided in the questionnaire.

### Biomarker analyses

Blood samples were collected after a 10- to 12-h overnight fast between December 2021 and June 2022, and serum or plasma was separated and stored at − 70 °C or − 80 °C until analysis. MicroVue enzyme-linked immunoassay kits (Quidel, San Diego, CA, USA) were used to analyse bone formation marker bone-specific alkaline phosphatase (BAP) and bone resorption marker tartrate-resistant acid phosphatase 5b (TRAP5b) concentrations in serum. Plasma intact PTH was analysed by immunochemiluminometric method with a Siemens Atellica® IM1600 analyser (Siemens, München, Germany). The S-25(OH)D concentrations were analysed using liquid chromatography-tandem mass spectrometry (LC–MS/MS). This LC–MS/MS method measures S-25(OH)D_2_ and S-25(OH)D_3_ separately, and total S-25(OH)D concentrations were calculated based on the sum of these values. The inter- and intra-CV for the analyses were < 5% and < 6%, respectively, for both metabolites [28]. The quality and accuracy of S-25(OH)D analysis by the LC–MS/MS in the laboratory are monitored on an ongoing basis by participation in the DEQAS (Charing Cross Hospital, London, UK)]. Serum Ca (S-Ca), phosphate (S-Pi), total alkaline phosphatase (S-ALP), and creatinine (used for eGFR calculation) concentrations were analysed using a photometric method with an Indiko automatic analyser (Thermo Clinical Labsystems Oy, Espoo, Finland). Intra-assay CVs were ≤ 3.7% for BAP, ≤ 3.1% for TRAP5b, 4% for PTH, ≤ 4.0% for S-Ca, S-Pi, serum creatinine, and S-ALP. Inter-assay CVs were 7.0% for BAP, 3.0% for TRAP5b, 6% for PTH, and < 1.7% for S-Ca, S-Pi, serum creatinine, and S-ALP. For adults, kidney function in terms of estimated glomerular filtration rate (eGFR) was calculated using the CKD-EPI formula [29] based on participant’s age and serum creatinine concentration.

### Nutrient intake analyses

For dietary data, FRs covering two predefined weekdays and for one weekend day were collected. Caregivers and early educators recorded children’s food intake on separate FR forms during the same day. Participants received written instructions for filling in the FR. Validated portion size estimation booklets for children and adults were provided [30–32], and a link to an instructional video was also made available. FRs were reviewed by a trained nutritionist upon return, and any missing information was requested from the participants. FRs for one child and one adult in the OMN groups were missing, and their data were only used in the biomarker analysis. Four children had incomplete FRs; for two children, complete data were available for two days, and for two children for one day; means of these days were used in the nutrient intake analysis. Missing FR days were mainly due to missing data at certain meals at ECEC centres.

Nutrient intakes were calculated using AromiDiet software (version 14.10.10.6, CGI Inc.), which is based on the Finnish food composition database Fineli® (release 20), administrated by the Finnish Institute for Health and Welfare. As the AromiDiet lacks comprehensive coverage of the current plant-based products, the nutritionists carefully complemented the database with new products and recipes. After extracting the data from the software, each food code (representing either a food item or a composite dish) was assigned to a food group and nutrient retention factors were applied using a single factor per nutrient per food group [33]. For this paper, daily means of energy, macronutrients (percentages of total energy), vitamin D, Ca, and phosphorus (P) intakes as well as calcium-to-phosphorus (Ca:P) molar ratio (mol/mol), and protein intakes per body weight were calculated. Daily Ca and vitamin D intakes from supplements (based on questionnaire data) were calculated and taken into account when total intakes for supplement users were reported.

### Statistical analyses

Statistical analyses were performed with SPSS Statistics version 29 (IBM, New York, NY, USA). Normality of the data was assessed using the Kolmogorov–Smirnov test. Variables were log10-transformed to improve normality, if needed. All biomarker variables, except TRAP5b and S-Ca, were log10-transformed. Data are presented as means and SD or SEM. All tests were considered significant at *P* < 0.05. Differences in bone turnover and mineral metabolism marker concentrations between the diet groups were analysed by ANOVA without adjustment and by ANCOVA adjusted for sex, age, and (SDS-)BMI. Post hoc comparisons between the diet groups were carried out with Bonferroni corrections. Contrast analyses were performed to test whether there are significant linear trends in diet group means of the biomarker concentrations towards more PBD, i.e. from mixed (OMN) through partly plant-based (VGT) to completely plant-based (VGN) diet, adjusted for the above-mentioned covariates. The results of the adjusted model are presented as the main results of this work. Month of blood sampling was also tested in total 25(OH)D and 25(OH)D_3_ analyses as a covariate (a continuous variable in accordance with increased sunlight in Finland towards summer months, Helsinki 60°N; [34]), but it did not affect the results. In addition, the percentages of subjects having deficient (S-25(OH)D < 30 nmol/l), inadequate (S-25(OH)D between 30 and 49.9 nmol/l), and adequate (S-25(OH)D ≥ 50 nmol/l) vitamin D status were calculated [1, 35]. For analysing differences in categorical variables, Chi-square tests were used.

Differences in nutrient intakes and background variables between the groups were mainly analysed by ANOVA; however, the non-parametric independent samples Kruskal–Wallis test was employed for vitamin D variables and molar Ca:P ratio. Post hoc comparisons between the diet groups were performed with Bonferroni corrections. All nutrient data, except intakes of energy-yielding nutrients (E%), protein per body weight (only adults), and vitamin D as well as Ca:P ratio, were log-transformed.

Potential medications affecting bone turnover were checked and analyses were also carried out without subjects using corticosteroids (n = 1) and isotreonine (n = 1), but these did not affect the results, nor did either of these participants have extreme values in any of the measured biomarkers, and thus, these subjects were included in the final analysis. Regarding eGFR, 24 adults (VGN n = 9, VGT n = 7, OMN n = 8) had eGFR below the age-specific reference limit, but as all had eGFR > 60 ml/(min*1.73 m^2^), these subjects were included in the data. No cases of severe hyperparathyroidism were observed (all subjects had P-PTH ≤ 91 ng/l).

## Results

### Background characteristics

Of the children, 51% were girls, with a mean age of 4.5 years (SD 1.5), and 89% had followed their VGN (including breastmilk) or VGT diet since birth (Table 1). Of the caregivers, 58% were women, with a mean age of 38.2 years (SD 4.4), and were highly educated, normal weight, moderately to highly physically active, and consumed alcohol very moderately (Table 1). The majority of the adults (86%) had followed their VGN or VGT diet for longer than two years at the time of data collection.

**Table 1:** Background characteristics of child (n = 74) and adult (n = 75) participants in the MIRA2 study stratified by diet group

	Vegan	Vegetarian	Omnivorous	*P*
Children (n = 74)	n = 29	n = 18	n = 27	
Sex (female n, %)	17 (58.6%)	8 (44.4%)	13 (48.1%)	0.586
Age (y)	4.5 (1.5)	4.5 (1.6)	4.4 (1.4)	0.995
Parental education level^1^				0.040
Low	1 (3.4%)	0 (0.0%)	2 (8.0%)	
Intermediate	5 (17.2%)	0 (0.0%)	10 (40.0%)	
High	23 (79.3%)	18 (100.0%)	13 (52.0%)	
Standardized body mass index	− 0.04 (0.84)	0.32 (1.04)	− 0.03 (1.12)	0.439
Followed the diet since birth (n/%)	25 (86.2%)	17 (94.4%)	na	na
Adults (n = 75)	n = 28	n = 23	n = 24	
Sex (female n, %)	18 (64.3%)	13 (56.5%)	12 (50.0%)	0.581
Age (y)	37.2 (3.7)	40.3 (4.6)	37.4 (4.5)	0.033*
Education level^2^				0.299
Low	4 (14.3%)	1 (4.3%)	3 (12.5%)	
Intermediate	6 (21.4%)	4 (17.4%)	9 (37.5%)	
High	18 (64.3%)	18 (78.3%)	12 (50.0%)	
Body mass index (kg/m^2^)	22.9 (2.8)	24.3 (3.6)	23.8 (1.9)	0.227
Everyday physical activity (n/%)^3^				0.416
Low	5 (17.9%)	1 (3.7%)	6 (25.0%)	
Moderate	12 (42.9%)	12 (52.2%)	9 (37.5%)	
High or very high	11 (39.3%)	10 (43.5%)	9 (37.5%)	
Smoking (n/%)				0.075
Never-smoker	21 (75.0%)	15 (65.2%)	22 (91.7%)	
Former smoker	6 (21.4%)	4 (17.4%)	2 (8.3%)	
Current smoker	1 (3.6%)	4 (17.4%)	0 (0.0%)	
Alcohol consumption (servings/month)^4^	3.9 (5.5)	6.5 (6.5)	5.1 (5.4)	0.203
Estimated glomerulus filtration rate (ml/min/1.73 m^2^)^5^	101.0 (16.5)	98.8 (12.5)	95.1 (15.2)	0.402
Contraceptive use (of women)	2 (11.1%)	2 (15.4%)	0 (0.0%)	0.392
Followed the diet since year (n/%)				na
2020–2022	3 (10.7%)	1 (4.3%)	na	
2015–2019	10 (35.7%)	4 (17.4%)	na	
2007–2015	9 (32.1%)	8 (34.8%)	na	
2006 or earlier	5 (17.9%)	8 (34.8%)	na	
No information	1 (3.6%)	2 (8.7%)	na	

Statistical test ANOVA, except for categorical variables Chi-square test

For age, alcohol consumption, and BMI, log10-transformed variables were used

Standard deviation score for body mass index was calculated based on Finnish population growth data [27], data missing for one child following an omnivorous diet

^1^Highest education in family, data missing for 2 omnivorous children. ^1,2^Education: low – comprehensive school, vocational school, upper secondary school; intermediate – bachelor’s degree or equivalent; high – master’s degree or higher

^3^Physical activity classes: Low = spending most of free time reading, watching TV, doing crafts, or other activities that require little physical activity, Moderate = walking, riding a bike, or doing moderately heart rate-raising household chores at least three times a week, High = engaging in physical activity that causes shortness of breath or heavy gardening and outdoor work for at least three hours a week, Very high = engaging in physical activity that causes shortness of breath or heavy gardening and outdoor work for at least five hours a week

^4^One alcohol serving (12 g of 100% alcohol), equivalent to 0.33 l medium-strength beer, 12 cl wine, or 4 cl spirits

^5^vegan n = 26, vegetarian n = 21, omnivorous n = 20

*post hoc comparison with Bonferroni correction, *P* > 0.05

Missing data for vitamin D supplement use: adults vegan n = 2, vegetarian n = 6, omnivorous n = 6; children omnivorous n = 2

### Bone turnover and mineral metabolism markers

#### Children

**Fig. 2:**
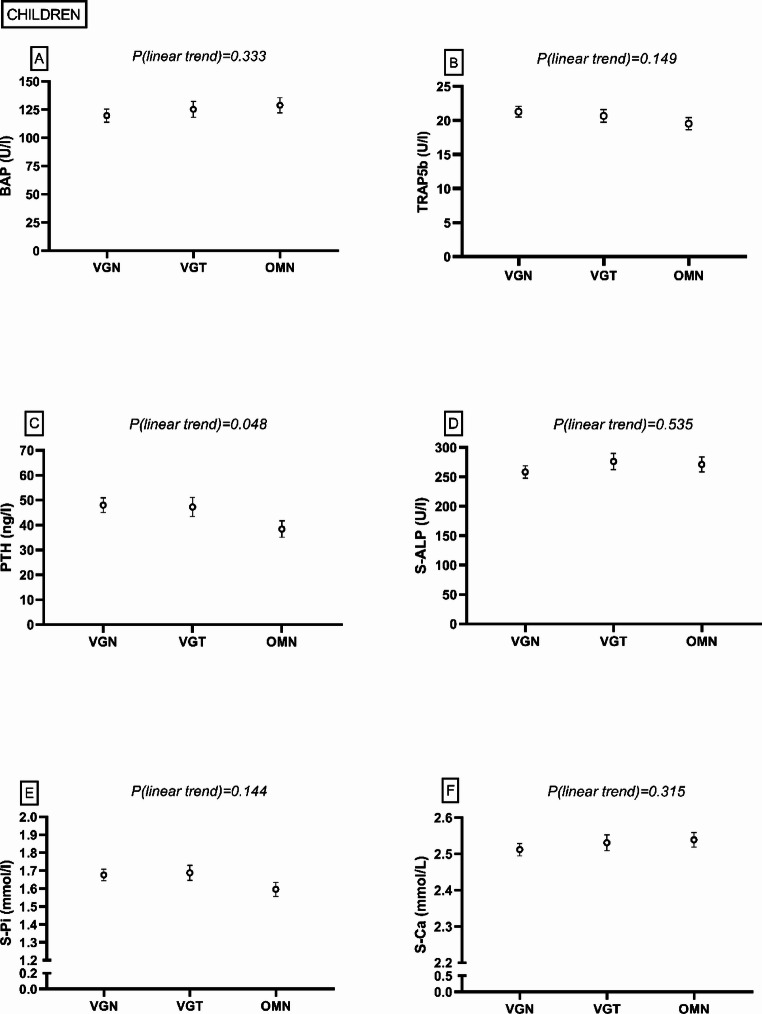
Bone and mineral metabolism markers among children following a vegan (VGN), vegetarian (VGT), or omnivorous (OMN) diet in the MIRA2 study. **A** serum bone-specific alkaline phosphatase (BAP), **B** serum tartrate-resistant acid phosphatase 5b (TRAP5b), **C** plasma parathyroid hormone (PTH), **D** serum total alkaline phosphatase (S-ALP), **E** serum phosphate (S-Pi), **F** serum calcium (S-Ca). Linear trend test performed by contrast analysis, adjusted for age, sex, standardized body mass index. VGN PTH n = 28, others n = 26; VGT TRAP5b, BAP n = 18, PTH n = 17, others n = 16; OMN PTH n = 22, BAP, TRAP5b n = 19, others n = 18

**Fig. 3:**
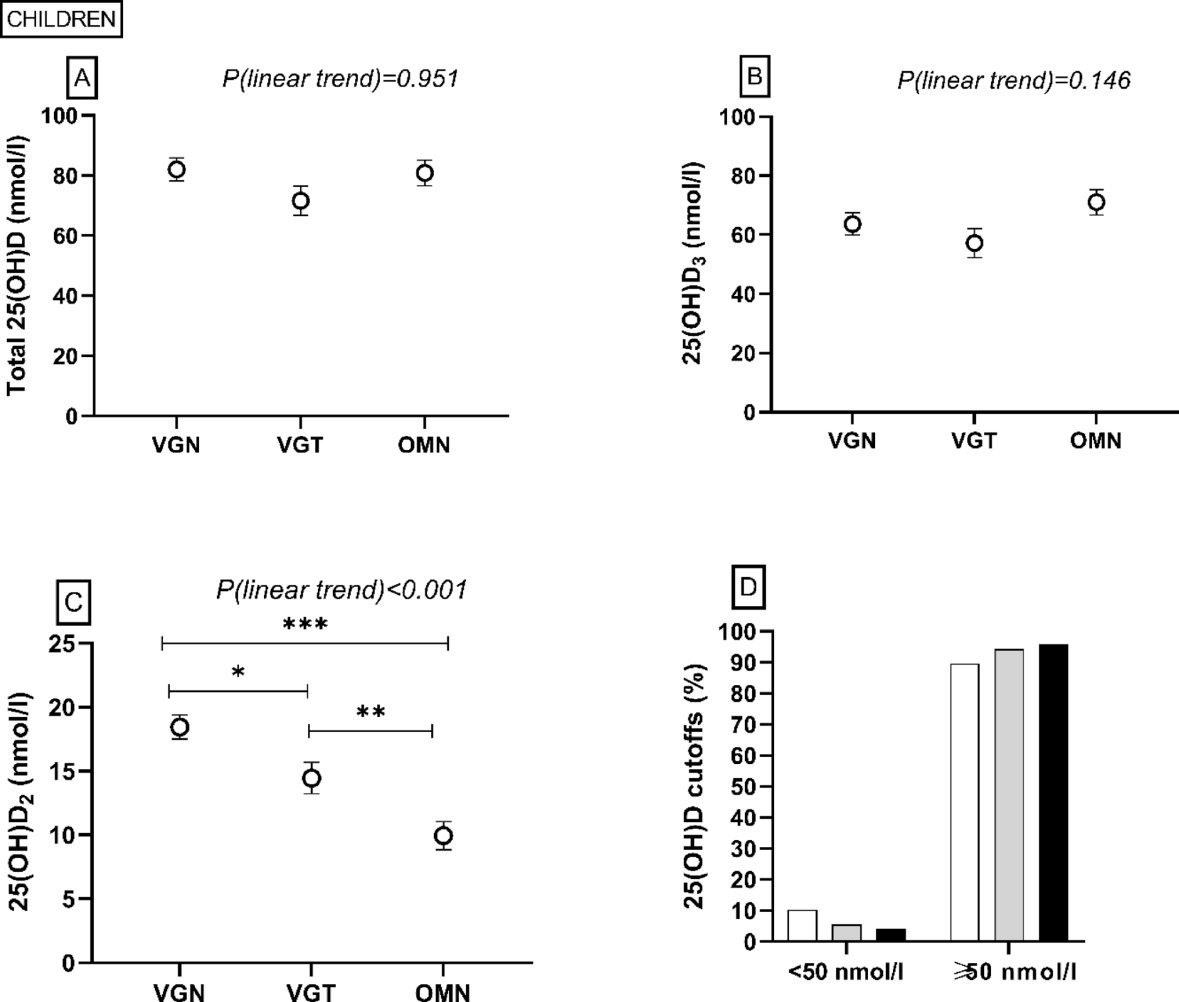
Vitamin D status among children following a vegan (VGN/white column, n=29), vegetarian (VGT/grey column, n=18), or omnivorous (OMN/black column, n=23) diet in the MIRA2 study. **A** serum total 25-hydroxyvitamin D (25(OH)D), **B** serum 25-hydroxyvitamin D_3_ (25(OH)D_3_), **C** serum 25-hydroxyvitamin D_2_, S-25(OH)D_2_, **D** percentages of subjects according to total 25(OH)D cut-offs < 50 nmol and ≥ 50 nmol/l [1]. Linear trend test performed by contrast analysis, adjusted for age, sex, standardized body mass index. *P < 0.05 **P < 0.01 ***P < 0.001 ANCOVA, adjusted for age, sex, standardized body mass index, post hoc analysis with Bonferroni correction

No differences in bone turnover markers TRAP5b and BAP were observed (*P* > 0.05), but there was a linear trend towards higher PTH in PBDs (i.e. going from OMN to VGT to VGN) (*P* = 0.048) (Fig. 2). Total 25(OH)D or 25(OH)D_3_ did not differ between the groups (*P* > 0.05) (Fig. 3). In the VGN group, 25(OH)D_2_ was higher than in the OMN or VGT group (P < 0.001, *P* = 0.034, respectively), and also the VGT group had higher 25(OH)D_2_ than the OMN group (*P* = 0.009). Most of the children had adequate vitamin D status (i.e. total 25(OH)D > 50 nmol/l) (Fig. 3). None of the children showed vitamin D deficiency (25(OH)D < 30 nmol/l), but three (10%) in the VGN group, and one in the VGT (6%) and OMN (4%) groups had inadequate vitamin D status (total 25(OH)D 30–49.9 nmol/l) [1, 35]. S-ALP, S-Pi, or S-Ca did not differ between the groups (*P* > 0.05) (Fig. 2). Supplemental Table 1 shows more detailed information.

#### Adults

**Fig. 4:**
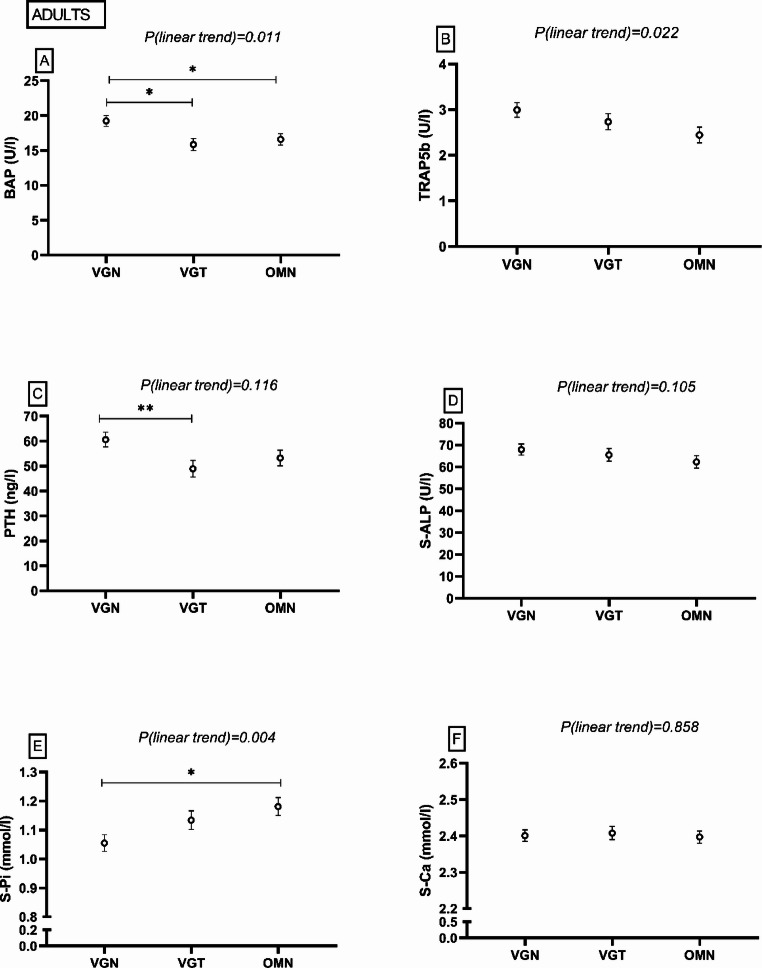
Bone and mineral metabolism markers among adults following a vegan (VGN), vegetarian (VGT), or omnivorous (OMN) diet in the MIRA2 study. **A** serum bone-specific alkaline phosphatase (BAP), **B** serum tartrate-resistant acid phosphatase 5b (TRAP5b), **C** plasma parathyroid hormone (PTH), **D** serum total alkaline phosphatase (S-ALP), **E** serum phosphate (S-Pi), **F** serum calcium (S-Ca). Linear trend test performed by contrast analysis, adjusted for age, sex, body mass index. *P < 0.05, **P < 0.01, ANCOVA, adjusted for age, sex, body mass index, post hoc analysis with Bonferroni correction. VGN PTH n = 28, S-Pi n = 25, others n = 26; VGT PTH n = 23, TRAP5b, BAP n = 22, others n = 21; OMN PTH n = 24, TRAP5b, BAP n = 23, others n = 21

**Fig. 5:**
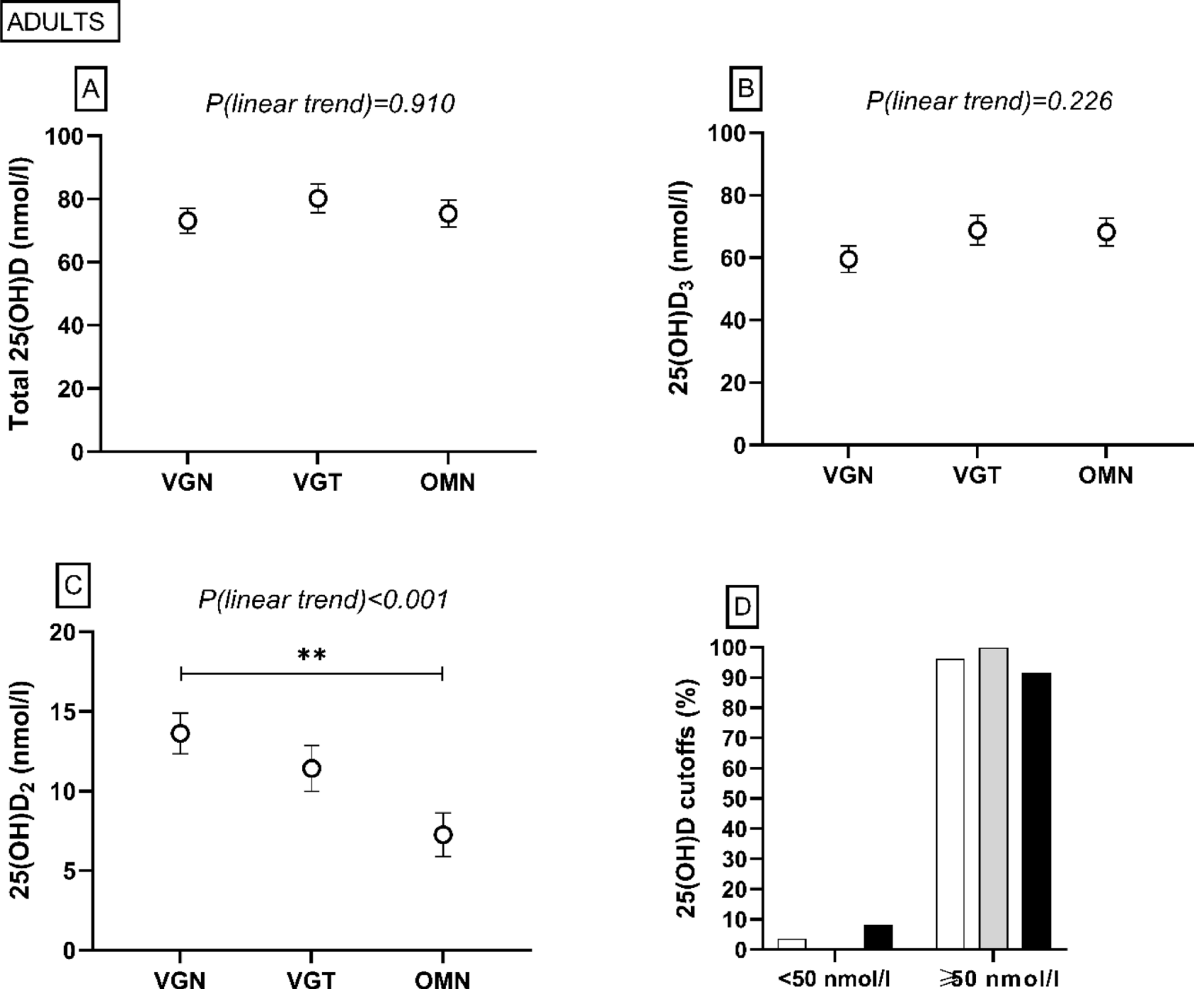
Vitamin D status among adults following a vegan (VGN/white column, n=28), vegetarian (VGT/grey column, n=23), or omnivorous (OMN/black column, n=24) diet in the MIRA2 study. **A** serum total 25-hydroxyvitamin D (25(OH)D), **B** serum 25-hydroxyvitamin D_3_ (25(OH)D_3_), **C** serum 25-hydroxyvitamin D_2_ (S-25(OH)D_2_), **D** percentages of subjects according to total 25(OH)D cut-offs < 50 nmol and ≥ 50 nmol/l [1]. Linear trend test performed by contrast analysis, adjusted for age, sex, body mass index **P < 0.01, ANCOVA, adjusted for age, sex, body mass index, post hoc analysis with Bonferroni correction

Bone formation marker BAP was higher in the VGN group than in the OMN and VGT groups (*P* = 0.034, *P* = 0.010), and a linear trend emerged for higher BAP in PBDs (*P* = 0.011) (Fig. 4). For bone resorption marker TRAP5b, there was a linear trend for higher TRAP5b in PBDs (*P* = 0.022). PTH was higher in the VGN group than in the VGT group (*P* = 0.005). S-Pi was lower in the VGN group than in the OMN group (*P* = 0.013). Total 25(OH)D or 25OHD_3_ did not differ between the groups (*P* > 0.05), but 25(OH)D_2_ was higher in the VGN group than in the OMN group (*P* = 0.003) (Fig. 5). Most of the adults had adequate vitamin D status, but one adult (4%) in the VGN group, and two adults (8%) in the OMN group had inadequate status [1, 35]. We did not observe any differences in S-ALP or S-Ca (*P* > 0.05) (Fig. 4). Supplemental Table 2 shows more detailed information.

### Energy, macronutrient, and bone-related nutrient intakes

Among children, carbohydrate (E%) or fat (E%) intakes did not differ between the groups (*P* > 0.05) but VGN and VGT groups had lower protein intakes (12.0 ± 1.8 E% and 14.3 ± 1.5 E%) and higher fibre intakes (3.7 ± 0.5 E% and 3.4 ± 0.8 E%) than the OMN group (protein 16.2 ± 2.3 E%, *P* ≤ 0.004; fibre 2.6 ± 0.5 E%, P < 0.001) (Table 2). Energy intakes were higher in the VGT group (5766 ± 875 kJ/d) than in the OMN group (5042 ± 1106 kJ/d) (*P* = 0.038). In adults, the trend in energy-yielding nutrients was similar, without differences in energy intakes (*P* > 0.05) (Table 3).

**Table 2:** Daily energy and nutrient intakes of child participants (n = 73) in the MIRA2 study stratified by diet group

	Vegan		Vegetarian		Omnivorous		*P*	P (Bonferroni correction)		
	n = 29		n = 18		n = 26					
	Mean	SD	Mean	SD	Mean	SD		VGN vs. VGT	VGN vs. OMN	VGT vs. OMN
Energy (kJ)	5615	1070	5766	875	5042	1106	0.0243	ns	ns	0.038
Protein (E%)	12.0	1.8	14.3	1.5	16.2	2.3	< 0.001	< 0.001	< 0.001	0.004
Protein (g/kg body weight)	2.36	0.61	2.56	0.65	2.77	0.60	0.040	ns	0.035	ns
Carbohydrates (E%)	52.0	5.4	50.0	4.4	49.5	4.9	0.150	–	–	–
Fat (E%)	32.2	3.9	32.0	4.5	32.2	4.5	0.986	–	–	–
Fibre (E%)	3.7	0.5	3.4	0.8	2.6	0.5	< 0.001	ns	< 0.001	< 0.001
Vitamin D										
Dietary (μg)	7.87	2.94	7.09	2.63	8.00	3.40	0.661	–	–	–
Dietary (μg/MJ)	1.40	0.41	1.23	0.40	1.60	0.66	0.155	–	–	–
Supplement users (n/%)*	29/100%		18/100%		23/92.0%		0.145			
Total (diet + supplement) (μg)**	26.8	10.5	18.0	3.9	18.0	4.7	< 0.001	< 0.001	< 0.001	ns
Supplemental (μg)**	18.9	10.3	10.9	3.5	10.5	4.5	< 0.001	0.001	< 0.001	ns
Calcium										
Dietary (mg)	761	256	851	224	782	289	0.457	–	–	–
Dietary (mg/MJ)	138	47	148	37	155	47	0.362	–	–	–
Supplement users (n/%)*	2/6.9%		2/11.1%		0/0%		na			
Total (diet + supplement) (mg)**	997	250	653	18	na	na	na	na	na	na
Supplemental (mg)**	143	0	188	88	na	na	na	na	na	na
Phosphorus (mg)	835	164	1018	175	998	280	0.006	0.011	0.032	ns
Phosphorus (mg/MJ)	150	22	177	20	198	38	< 0.001	0.002	< 0.001	ns
Calcium-to-phosphorus ratio (mol/mol)	0.70	0.17	0.64	0.12	0.60	0.11	0.039	ns	0.033	ns

*VGN* vegan, *VGT* vegetarian, *OMN* omnivorous

Log10-transformed variables were used for other than energy-yielding nutrients (except protein per body weight), vitamin D variables, and calcium-to-phosphorus ratio

Statistical tests performed with ANOVA, except for vitamin D variables and calcium-phosphorus ratio non-parametric independent samples Kruskal–Wallis test was used

Vitamin D supplement use proportions were analysed by Chi-square tests

*Percentages calculated based on n = 72 (OMN n = 23, data for 2 subjects missing). **Intakes presented for supplement users only

*na* not applicable, *ns* non-significant

**Table 3:** Daily energy and nutrient intakes of adult participants (n = 74) in the MIRA2 study stratified by diet group

	Vegan		Vegetarian		Omnivorous		P	P (Bonferroni correction)		
	n = 28		n = 22		n = 24					
	Mean	SD	Mean	SD	Mean	SD		VGN vs. VGT	VGN vs. OMN	VGT vs. OMN
Energy (kJ)	8581	2085	8616	1993	8767	2512	0.968	–	–	–
Protein (E%)	13.5	2.2	14.1	1.8	17.1	3.3	< 0.001	ns	< 0.001	< 0.001
Protein (g/kg body weight)	1.03	0.29	0.96	0.17	1.23	0.36	0.006	ns	0.040	0.007
Carbohydrates (E%)	45.4	6.6	42.1	7.4	41.1	7.3	0.078	–	–	–
Fat (E%)	35.8	6.8	38.5	6.8	38.8	5.5	0.173	–	–	–
Fibre (E%)	3.5	0.7	3.2	0.8	2.1	0.7	< 0.001	ns	< 0.001	< 0.001
Vitamin D										
Dietary (μg)	6.50	4.43	6.22	4.23	8.61	4.70	0.101	–	–	–
Dietary (μg/MJ)	0.75	0.44	0.73	0.47	0.98	0.48	0.081	–	–	–
Supplement users (n/%)*	26/92.9%		17/73.9%		18/75.0%		0.141	–	–	–
Total (diet + supplement) (μg)**	50.0	25.6	37.8	27.2	43.9	36.5	0.092	–	–	–
Supplemental (μg)**	43.3	24.8	30.9	28.3	34.8	37.4	0.040	–	–	–
Calcium										
Dietary (mg)	920	334	1055	234	1015	423	0.252	–	–	–
Dietary (mg/MJ)	110	36	127	33	114	32	0.188	–	–	–
Supplement users (n/%)*	10/35.7%		4/17.4%		2/8.3%		na	na	na	na
Total (diet + supplement) (mg)**	1093	387	1277	197	1677	1224	na	na	na	na
Supplemental (mg**)	219	220	201	210	750	354	na	na	na	na
Phosphorus (mg)	1319	326	1404	329	1516	545	0.349	–	–	–
Phosphorus (mg/MJ)	155	25	164	23	171	25	0.068	–	–	–
Calcium-to-phosphorus ratio (mol/mol)	0.54	0.16	0.60	0.12	0.52	0.11	0.089	–	–	–

*VGN* vegan, *VGT* vegetarian, *OMN* omnivorous

Log10-transformed variables were used for other than energy-yielding nutrients, vitamin D variables, and calcium-to-phosphorus ratio

Statistical tests performed with ANOVA, except for vitamin D variables and calcium-phosphorus ratio non-parametric independent samples Kruskal–Wallis test was used

Vitamin D supplement use proportions were analysed by Chi-square tests

*Percentages calculated based on n = 75 (VGT n = 23 including 1 subject who had biomarker but not dietary data)

**Intakes presented for supplement users only, for calcium only intake from supplements > 15 mg/d included

*na* not applicable, *ns* non-significant

There were no differences in vitamin D intake from food across the diet groups in children (*P* > 0.05) (Table 2) or in adults (*P* > 0.05) (Table 3). Mean intakes from food were below the recommended intake (RI) (10 µg/d) [1], but supplement use was widespread, with 97.2% of children and 84.0% of adults taking vitamin D supplements. Among supplement users, mean total vitamin D intakes were adequate in all age and diet groups. Children in the VGN group had higher total (26.8 ± 10.5 µg/d) and supplemental vitamin D (18.9 ± 10.3 µg/d) intakes than children in the OMN (18.0 ± 4.7 µg/d, 10.9 ± 3.5 µg/d, respectively) and VGT (18.0 ± 3.9 µg/d, 10.5 ± 4.5 µg/d, respectively) groups (*P* ≤ 0.001). On average, the supplement doses exceeded recommended doses (children 7.5 µg/d, adults 10 µg/d) [24].

All supplements used by children were D_3_ supplements. Among adults, one VGT participant used vitamin D_2_ supplements, one VGN participant used both D_2_ and D_3_ supplements, and all others used vitamin D_3_ supplements. Total vitamin D intakes of two children following a VGN diet exceeded the tolerable upper intake level (UL) of 50 µg/d (intakes 56–58 µg/d) [36]. Among adults, the UL of 100 µg/d was exceeded by one VGN, one VGT, and three OMN participants (intakes 106–127 µg/d) [36]. Exceeding the UL was primarily due to supplement use.

Dietary Ca intakes did not differ between the diet groups in either children (*P* > 0.05) (Table 2) or adults (*P* > 0.05) (Table 3). On average, Ca intakes were adequate and above AR and RI (adults: AR 750 mg/d, RI 950 mg/d; children AR 395–675 mg/d, RI 450–850 mg/d, depending on age group) [1] except the VGN adult group had intakes slightly below RI (920 ± 334 mg/d). Of children, 6% and of adults 17% used Ca or Ca-containing multivitamin supplements. Among children the VGN group had a higher Ca:P ratio (0.70 ± 0.17) than the OMN group (0.60 ± 0.11; *P* = 0.033) whereas among adults the ratio or P intakes did not differ (*P* > 0.05). Among children, P intake, both in absolute terms (835 ± 164 mg/d) and per megajoule (150 ± 22 mg/MJ/d), was lowest in the VGN group (*P* ≤ 0.032).

## Discussion

Here, in a cross-sectional study, we report bone and mineral metabolism and intakes of bone-related nutrients in 2–7-year-old children and their adult caregivers following VGN, VGT, and OMN diets. We found signs of increased bone catabolism in PBD groups among children, indicated by higher PTH concentrations, while among adults following PBDs signs of accelerated bone turnover, evidenced by higher concentrations of the bone resorption marker TRAP5b and the bone formation marker BAP, were present. These findings were not explained by the vitamin D status that was adequate (> 50 nmol/l) in most adults and children. Also Ca intakes were similar between the groups but protein intakes (E% and per body weight) were lower in both children and adults following PBDs.

Ca and vitamin D have been considered as the most important bone-related nutrients, but adequate protein intake is also essential for maintaining bone health throughout life [37]. At a mechanistic level, dietary protein has been suggested to participate in bone mineralization by enhancing intestinal Ca absorption and increasing circulating concentrations of insulin-like growth factor 1, causing stimulation of osteoblastic function and bone formation [38]. Although mean protein intakes in the current study met the recommendations (10–20 E%) [1], participants following a VGN diet in both age groups had the lowest protein intakes (12.0–13.5 E%), while the highest intakes were observed in those following the OMN diet (16.2–17.1 E%), trends being similar concerning protein intake per body weight. Since plant proteins have lower quality than animal proteins in terms of lower digestibility and poorer amino acid composition [39], in our study population, where mean vitamin D status was adequate, and mean Ca intakes above or close to the RI, the role of protein may be more pronounced.

Compared with the results of earlier studies on Finnish children and adults following VGN diets [26, 40], the total 25(OH)D concentrations in our study were higher and most of the subjects had adequate 25(OH)D concentration, probably as a consequence of frequent vitamin D supplement use and wider vitamin D fortification of plant-based milk alternatives. Finland is one of the few countries where a wide national vitamin D food fortification policy has been implemented [25]. The voluntary fortification covers fluid milk products and fat spreads [23], and today most plant-based milk alternatives are also fortified. In addition, vitamin D supplementation guidelines target all children, people following a VGN diet, and those who do not regularly consume fortified products or fish [24, 41]. All children and the majority of the adults in the VGN group in our study used vitamin D supplements. Total vitamin D intake among supplement users was in line with recommendations (10 µg/d) [24]. However, some reported supplementation doses were high and exceeded the age-specific UL [36]. All participants following a VGN diet used vitamin D_3_ supplements (vitamin D originating from algae), the form considered more effective in raising 25(OH)D concentrations than vitamin D_2_ [42]. Thus, our current study showed that the Finnish fortification and supplementation strategies are an effective way to increase vitamin D intake.

We observed lower S-Pi concentrations among adults following a VGN diet than among those following an OMN diet, even though P intakes or dietary molar Ca:P ratios did not differ between the groups. In children, however, S-Pi was similar within the groups, despite the VGN group having lower P intakes and higher Ca:P ratios than the OMN group. Lower Ca:P ratio has been shown to be harmful for bone even when Ca intake is adequate [43]. However, for some reason, a similar relation was not observed in the current study. Most of the plant-based milk and yoghurt alternatives on the Finnish market are also fortified with Ca, which was reflected in our results as the Ca intakes were approximately adequate and in line with findings of previous Finnish studies on children and adults following VGN diets [26, 40]. Nevertheless, Ca bioavailability in plant-based foods varies: it is generally lower than in the dairy products, and the presence of antinutrients such as phytate or oxalate impairs Ca bioavailability [44]. Moreover, in vitro studies have shown lower Ca bioaccessibility in plant-based products fortified with Ca triphosphate compared to those fortified with Ca carbonate [45]. Thus, lower Ca bioavailability in the PBDs, especially among VGN groups, may play a role in our findings. We observed no differences in the S-Ca concentrations, but these are tightly regulated and therefore may not reflect differences in diets.

The fact that children showed no differences in bone formation or resorption markers while among adults signs of such were visible, may be due to the different shift of bone turnover during childhood and adulthood [46]. In early childhood, the dominating stage is modelling, characterized by bone formation and shaping of the bone, whereas for adults the typical process is bone remodelling: old bone is replaced or renewed. Previous studies focused on bone turnover in 4–10-year-old Polish children comparing VGT (n = 44–70) and OMN (n = 24–60) diets [18–21]. These studies mainly reported higher bone resorption marker [18, 20, 21] and PTH concentrations [19] associating especially on VGT diets but results for bone formation markers were inconsistent [18, 19, 21]. Moreover, studies have shown lower BMD or BMC in children following PBDs [18, 22]. Furthermore, in addition to higher bone resorption, amino acid intakes and serum concentrations of some essential amino acids were lower in VGT despite the diet having adequate (13 E% but still lower than that of OMN; 16 E%) protein intakes [20]. Our results together with previous literature raise a question whether protein quality affects bone health.

Higher concentrations of bone resorption and/or formation markers have systematically been observed in VGN adults relative to OMN adults [14–16], which are in line with our results. However, we did not find any differences in S-ALP that can be originated in addition to bone from e.g. liver or intestine. As vitamin D and PTH generally interact, and PTH rises in response to lower vitamin D status [8], our results of VGN adults having higher PTH concentrations than VGT adults, but PBD groups not differing from OMN, are slightly surprising since the vitamin D status in all groups was adequate. Overall, previous studies have mainly shown higher PTH concentrations in VGN than OMN [14–16, 47] or VGT [47], two of these despite of similar vitamin D status [15, 16]. In our previous randomized clinical intervention, we did not find any differences in bone turnover using the same biomarkers, TRAP5b and BAP, as in this study, after a partial replacement of red and processed meat with non-soy legumes for six weeks (20% of total protein intake originated from legumes instead of red and processed meat) in adult men [48]. However, in both the meat-rich group and the legume-rich groups, 25(OH)D decreased and PTH increased, while no differences in Ca or vitamin D intakes were observed since neither legumes nor meat are their major source. In our other trial, we observed accelerated bone turnover after 12 weeks on a diet in which 70% of total protein intake originated from plant-based sources compared with a diet where 70% of total protein intake was of animal origin, which was most likely due to lower intakes of Ca and vitamin D, as also the amount of dairy products was decreased [49]. Our present results in combination with the previous ones stress the importance of versatile, well-planned diets that provide adequate amounts of the nutrients important for bone, regardless of the type of flexitarian or PBD.

### Strengths and limitations

To our knowledge, this is the first study reporting bone turnover in families following PBD, particularly in a country where strong national policies exist to improve vitamin D status. As a result, our findings may differ from studies conducted in countries without such strong vitamin D policies, where the lower availability of fortified plant-based dairy alternatives and supplementation practices could contribute to poorer vitamin D status in individuals following PBDs. Nevertheless, the dietary data were collected carefully, addressing the gaps in the food composition data of plant-based products. However, a limitation is that we did not reach the targeted number of participants, which reduces the power of the analyses; for some variables, we were able to see linear trends but not statistically significant differences between the groups. Most participating families had at least one person with an academic degree, potentially making them more health-conscious and more likely to adhere to the current vitamin D supplementation guidelines than less educated families, thereby decreasing the generalizability of the results. Additionally, the cross-sectional design and absence of measurements of the BMC and BMD do not allow evaluation of causality between the PBD and bone health. The physical activity of caregivers was assessed only by a questionnaire. An accelometer-based measurement, assessing also physical activity of both the children and adults, would have improved the accuracy, being, however, more burdensome for participants.

## Conclusions

In this cross-sectional study of 2–7-year-old children and their caregivers, we found linear trends towards increased bone catabolism, indicated by higher PTH concentrations among children following PBDs, and accelerated bone turnover, evidenced by increased resorption and formation marker concentrations among adults following PBDs. These trends were observed despite adequate vitamin D status and approximately adequate Ca intakes in all diet and age groups, but protein intakes were lower in PBDs. The results highlight the need for further investigation on the role of lower, albeit adequate, protein intake and well as Ca bioavailability in the context of PBDs and bone health.

## Electronic supplementary material

Below is the link to the electronic supplementary material.

Supplementary Material 1

## Abbreviations

AR: Average requirement
BMC: Bone mineral content
BMD: Bone mineral density
BMI: Body mass index
Ca: Calcium
Ca:P ratio: Calcium-to-phosphorus ratio
CTX: C-terminal telopeptide of type I collagen
ECEC: Early childhood care and education
eGFR: Estimated glomerular filtration rate
FR: Food record
NNR: Nordic Nutrition Recommendations
OMN: Omnivorous diet
P: Phosphorus
PBD: Plant-based diet
PTH: Parathyroid hormone
RI: Recommended intake
S-25(OH)D: Serum 25-hydroxyvitamin
S-25(OH)D_2_: Serum 25-hydroxyvitamin D_2_
S-25(OH)D_3_: Serum 25-hydroxyvitamin D_3_
S-ALP: Serum total alkaline phosphatase
S-BAP: Serum bone-specific alkaline phosphatase
S-Ca: Serum calcium
SDS-BMI: Standard deviation score for body mass index
S-Pi: Serum phosphate
S-TRAP5b: Serum tartrate-resistant acid phosphatase 5b
VGN: Vegan diet
VGT: Vegetarian diet
