# Supplementary material for: Bone and mineral metabolism in 2–7-year-old Finnish children and their caregivers following vegan, vegetarian, and omnivorous diets
Source: Eur J Nutr. 2025 Sep 11;64(6):276. doi: 10.1007/s00394-025-03758-y (PMC12426156; doi:10.1007/s00394-025-03758-y)
Supplement: Supplementary file 1 — Supplementary Material 1 [file 394_2025_3758_MOESM1_ESM.pdf]

Supplementary Information

Supplemental Table 1. Bone turnover and mineral metabolism markers of children (n=71) following vegan, vegetarian and omnivorous diets, unadjusted and adjusted results.

		Vegan n=29[1]		Vegetarian n=18[2]		Omnivorous n=24[3]			P (Bonferroni correction)			
		mean	sd[4]/sem[5]	mean	sd[4]/sem[5]	mean	sd[4]/sem[5]	P ANOVA[4]/ ANCOVA[5]	VGN vs VGT	VGN vs OMN	VGT vs OMN	P for trend by contrast analysis
Serum tartrate-resistant acid phosphatase 5b (U/l)	unadjusted	21.5	4.47	20.4	3.83	19.6	3.57	0.312	1	0.405	1	0.135
	adjusted	21.3	0.78	20.7	0.93	19.5	0.91	0.348	1	0.448	1	0.149
Serum bone-specific alkaline phosphatase (U/l)	unadjusted	121	29.0	124	24.7	129	32.7	0.688	1	1	1	0.400
	adjusted	120	5.8	125	7.0	129	6.7	0.575	1	1	1	0.333
Plasma parathyroid hormone (ng/l)	unadjusted	48.3	17.2	47.7	19.0	37.0	10.9	0.037	1	0.049	1	0.016
	adjusted	48.0	2.97	47.2	3.86	38.4	3.30	0.117	1.000	0.143	1.000	0.048
Serum alkaline phosphatase (U/L)	unadjusted	260	48.0	273	55.4	271	60.1	0.755	1	1	1	0.599
	adjusted	258	10.7	276	13.8	271	12.9	0.612	1.000	1	1.000	0.535
Serum phosphate (mmol/l)	unadjusted	1.68	0.18	1.69	0.15	1.60	0.13	0.198	1	0.327	0.307	0.128
	adjusted	1.68	0.03	1.69	0.04	1.60	0.04	0.223	1.000	0.432	1.00	0.144
Serum calcium (mmol/l)	unadjusted	2.51	0.10	2.53	0.07	2.54	0.07	0.647	1	1	1	0.353
	adjusted	2.51	0.02	2.53	0.02	2.54	0.02	0.572	1	0.944	1	0.315
Serum total 25-hydroxyvitamin D (nmol/l)	unadjusted	82.0	23.0	72.4	14.5	80.9	19.4	0.357	0.566	1	0.647	0.974
	adjusted	82.1	3.75	71.6	4.82	80.9	4.24	0.299	0.446	1	0.565	0.951
Serum 25-hydroxyvitamin D_3 (nmol/l)	unadjusted	63.6	22.9	57.7	13.7	71.0	20.0	0.125	1	0.358	0.17	0.119
	adjusted	63.7	3.80	57.2	4.88	71.0	4.29	0.140	1	0.439	0.176	0.146
Serum 25-hydroxyvitamin D_2 (nmol/l)	unadjusted	18.4	5.30	14.7	6.10	9.92	4.48	<0.001	0.061	<0.001	0.006	<0.001
	adjusted	18.5	0.95	14.5	1.22	9.95	1.08	<0.001	0.034	<0.001	0.009	<0.001

VGN vegan, VGT vegetarian, OMN omnivorous; [1] n=28 for PTH, n=26 for TRAP5b, BAP, ALP, S-Ca, S-Pi; [2] n=17 for PTH, n=16 for ALP, S-Ca, S-Pi; 3 n=23 for 25(OH)D markers and PTH in adjusted models, n=19 for TRAP5b, BAP; n=18 ALP, S-Ca, S-Pi; [4] Unadjusted (P value from ANOVA, sd used for deviation); [5] Adjusted for age, sex, BMI (P value from ANCOVA, sem used for deviation)

Supplemental Table 2. Bone turnover and mineral metabolism markers of the adult participants (n=75) following vegan, vegetarian and omnivorous diet, unadjusted and adjusted results.

		Vegan n=28[1]		Vegetarian n=23[2]		Omnivorous n=24[3]		P	P (Bonferroni correction)			
		mean	sd[4]/sem[5]	mean	sd[4]/sem[5]	mean	sd[4]/sem[5]	[4]ANOVA/ [5]ANCOVA	VGN vs VGT	VGN vs OMN	VGT vs OMN	P for trend by contrast analysis
Serum tartrate-resistant acid phosphatase 5b (U/l)	unadjusted	2.96	1.19	2.70	0.73	2.51	0.68	0.288	0.973	0.268	1	0.089
	adjusted	3.00	0.16	2.74	0.18	2.45	0.17	0.070	0.876	0.065	0.758	0.022
Serum bone-specific alkaline phosphatase (U/l)	unadjusted	18.5	4.88	16.3	4.56	16.9	5.39	0.245	0.365	0.594	1	0.198
	adjusted	19.2	0.78	15.8	0.86	16.6	0.82	0.006	0.01	0.034	1	0.011
Plasma parathyroid hormone (ng/l)	unadjusted	59.6	16.6	48.9	15.5	52.4	14.9	0.049	0.047	0.444	0.969	0.148
	adjusted	60.6	2.95	46.9	3.31	53.2	3.15	0.007	0.005	0.348	0.260	0.116
Serum alkaline phosphatase (U/L)	unadjusted	66.1	13.9	67.4	17.6	62.8	15.7	0.620	1	1	1	0.402
	adjusted	68.0	2.54	65.5	2.89	62.3	2.81	0.262	1.000	0.316	1.000	0.105
Serum phosphate (mmol/l)	unadjusted	1.06	0.15	1.14	0.13	1.17	0.15	0.021	0.16	0.023	1	0.008
	adjusted	1.06	0.03	1.13	0.03	1.18	0.03	0.014	0.188	0.013	1.00	0.004
Serum calcium (mmol/l)	unadjusted	2.40	0.09	2.41	0.08	2.41	0.09	0.932	1	1	1	0.761
	adjusted	2.40	0.02	2.41	0.02	2.40	0.02	0.915	1	1	1	0.858
Serum total 25-hydroxyvitamin D (nmol/l)	unadjusted	73.7	17.2	79.8	19.8	75.1	24.3	0.499	0.92	1	0.903	0.959
	adjusted	73.1	4.01	80.2	4.50	75.4	4.28	0.517	0.859	1	1.000	0.91
Serum 25-hydroxyvitamin D_3 (nmol/l)	unadjusted	60.2	18.4	68.7	21.9	67.4	25.0	0.365	0.532	0.946	0.532	0.315
	adjusted	59.5	4.22	68.8	4.74	68.2	4.50	0.343	0.601	0.678	1	0.226
Serum 25-hydroxyvitamin D_2 (nmol/l)	unadjusted	13.5	6.47	11.2	6.09	7.65	7.50	0.009	0.808	0.007	0.154	0.002
	adjusted	13.6	1.29	11.4	1.45	7.25	1.38	0.004	0.814	0.003	0.095	<0.001

VGN vegan, VGT vegetarian, OMN omnivorous; 1 n=26 for TRAP5b, BAP, ALP, S-Ca, n=25 for S-Pi; [2] n=22 for TRAP5b, BAP, n=21 ALP, S-Ca, S-Pi; [3] n=23 for TRAP5b, BAP, n=21 ALP, S-Ca, S-Pi
[4] Unadjusted (P value from ANOVA, sd used for deviation); [5] Adjusted for age, sex, BMI (P value from ANCOVA, sem used for deviation)
